# Notes and News

**Published:** 1891-02-28

**Authors:** 


### Notes and News.

Collected and Communicated.

The Zenana Bible and Medical Mission has received intelligence from India, that the foundation stone of the new Hospital for Women, at Lucknow, was laid by Sir Auckland Colvin, Lieutenant-Governor of the North-West Provinces, on February 2nd, in the presence of a large number of European and native residents. The hospital is being built as a memorial to the late Dowager Lady Kinnaird, founder and late President of the Society.

A "voluntary collector" writes: "Agricola's" fair and reasonable comments on the Hospital Saturday Fund disagreement in your issue of February 21st will be received with satisfaction by the moderate members of the fund; but why does he use the figures £10,000? By the exertions of the paid canvassers (miscalled organising and assistant secretaries) the fund has been considerably raised. The figures are as follows: Progress of Fund—Total collection in 1888, £11,764; 1889, £13,927; 1890, £20,232.

At a meeting in the Manchester Town Hall the Mayor distributed St. John Ambulance certificates to upwards of sixty members of the city police force. In a brief address to the men, the Mayor stated that the Watch Committee were so much impressed with the importance of the knowledge of ambulance work by policemen, that they intended to make it almost, if not absolutely, a condition that men joining the force should qualify themselves by a course of training, to render what is known as "first aid" to the injured. At the present moment certificates are held by 385 men, which is a satisfactory proportion of the whole force.

The annual general Court of Governors of the Charing Cross Hospital was held on February 18th, in the board-room of the Hospital; Mr. T. B. Martin, the Treasurer, in the chair. The Secretary (Mr. A. E. Reade) read the seventieth annual report of the Council, from which it appeared that the number of in-patients had reached 2,165, and that the out-patients numbered 21,640, a total increase of over 400 from the previous year. As regarded finance, there had been a falling-off in donations, and the amount of legacies was the smallest in the history of the present hospital. Stock had to be sold to the amount of £3,662, and the bankers were further creditors to the amount of £3,000. The annual subscription list showed a very slight increase, as did the share due to the hospital of the medical school fees. A small staff of private nurses had been formed, but want of accommodation prevented increasing the staff sufficiently to meet the demands made upon it. It was announced that the triennial festival dinner would be held this year, when an earnest appeal for support and relief from financial anxieties would be moved. The adoption of the report was moved by the Chairman, and seconded by Mr. T. Hogg, and unanimously passed. The proceedings closed with the usual votes of thanks.

On Tuesday, March 3rd, Mrs. Calverley Bewicke will give one of her charming dramatic recitals in the Westminster Town Hall, in aid of the Samaritan Fund of the Westminster Hospital. The chair will be taken by the Ven. Archdeacon Farrar.

A scheme is on foot to form a Home for Epileptic Girls, in connection with the Brabazon Home of Comfort, Reigate, for members of the G. F. S. The Samaritan Branch of the National Hospital for Epilepsy is, we learn, working with the same object. It seems a pity that the two institutions should not work together in this matter, which creates a widespread interest. It would surely be to the advantage of the scheme to do so, and one of the homes might then be for men, instead of both being for girls, as is the present idea.

On February 10th the Seamen's Hospital Society held their annual court in the board-room of the P. and O. Company. Sir Thomas Sutherland took the chair, and there was a good attendance. The report was excellent, and referred specially to the work of the new branch hospital at the Albert Docks, and two of the speakers—Lord Brassey and the Bishop of Colchester—also made special mention of the "Baby Dreadnought." Over 12,000 seamen of all nations were treated by the Society last year, and the Austro-Hungarian Consul and the Danish Envoy, both bore testimony to the excellence of the institution. The Society has to raise £10,000 annually by subscriptions and donations.

The ladies of Lyme Regis have devised an excellent occupation for the time which lack of social duties leaves free during Lent. They have formed a sewing party to make warm and useful garments for the patients in their hospital, which can, if needful, be given to them when they leave. We hope this plan will recommend itself to others, as clothes of all kinds are always very welcome in the hospitals, where patients frequently arrive in clothes in such a condition that they can only be burnt. It is to meet the needs of such patients when they leave the hospital that Samaritan Funds are formed, but unfortunately these, as a rule, are very inadequately supported.

The annual meeting of the Governors of the Essex and Colchester Hospital was held on the 12th inst. Mr. H. G. Egerton presided. The report, which was satisfactory in every way, was taken as read. In moving its adoption, the Chairman said that the Committee had to mourn the loss of several friends of the hospital during the year, amongst whom was their Matron, who died in February. No legacies had fallen to the hospital, but they had had two very handsome donations, for which they returned grateful thanks. The hospital lived, as it were, upon the Hospital Saturday and Sunday collections, and if these were to fall off, their balance-sheet would bear a very different aspect. Lieut.-Colonel Holroyd seconded the adoption of the report, and after re-electing the officers, the proceedings terminated.

The greater part of St. Mary's Roman Catholic Hospital, Rochester, New York State, has been destroyed by a fire which took place on the night of the 15th inst. There were 250 patients and 19 nuns in the building at the time, and the flames spread very rapidly, yet all escaped, a fact which speaks volumes for the courage and presence of mind of the authorities and nurses, for many of the patients were unable to walk, and must inevitably have perished but for the nurses and others who carried them in their arms to a place of safety. The patients who were strong enough crowded to the windows, and were rescued by the firemen, who had early arrived on the scene. The eastern wing was entirely destroyed, and damage to the extent of £20,000 has been done. Mother Hieronyma, who founded the hospital, had that day completed her fiftieth year as a professed nun.

